# Association Between Cerebral Hypoperfusion and Cognitive Impairment in Patients With Chronic Vertebra-Basilar Stenosis

**DOI:** 10.3389/fpsyt.2018.00455

**Published:** 2018-09-26

**Authors:** Yiming Deng, Luyao Wang, Xuan Sun, Lian Liu, Meifang Zhu, Chunxue Wang, Binbin Sui, Mi Shen, Weibin Gu, Dapeng Mo, Ning Ma, Ligang Song, Xiaoqing Li, Xiaochuan Huo, Zhongrong Miao, Duanduan Chen, Feng Gao

**Affiliations:** ^1^Department of Interventional Neuroradiology, Beijing Tiantan Hospital, Capital Medical University, Beijing, China; ^2^China National Clinical Research Center for Neurological Diseases, Beijing, China; ^3^Center of Stroke, Beijing Institute for Brain Disorders, Beijing, China; ^4^Intelligent Robotics Institute, School of Mechatronical Engineering, Beijing Institute of Technology, Beijing, China; ^5^Departments of Neuropsychiatry and Clinical Psychology, Beijing Tiantan Hospital, Capital Medical University, Beijing, China; ^6^Department of Radiology, Beijing Tiantan Hospital, Capital Medical University, Beijing, China; ^7^School of Life Science, Beijing Institute of Technology, Beijing, China; ^8^Key Laboratory of Convergence Medical Engineering System and Healthcare Technology, The Ministry of Industry and Information Technology, Beijing Institute of Technology, Beijing, China

**Keywords:** vertebra-basilar stenosis, cognitive impairment, cerebral hypoperfusion, cerebral infarction, stroke

## Abstract

**Objective:** This study aimed to investigate the association between cognitive impairment and cerebral haemodynamic changes in patients with chronic vertebra-basilar (VB) stenosis.

**Methods:** Patients with severe posterior circulation VB stenosis and infarction or a history of infarction for more than 2 weeks from January 2014 to January 2015 were enrolled (*n* = 96). They were divided into three groups, namely, the computed tomography perfusion (CTP) normal group, the CTP compensated group, and the CTP decompensated group. Cognitive function was assessed using a validated Chinese version of the Mini-Mental State Examination (MMSE), the Frontal Assessment Battery (FAB), and the Repeatable Battery for the Assessment of Neuropsychological Status (RBANS). Regression models were used to identify independent risk factors for cognitive impairment.

**Results:** The MMSE and FAB scores of patients in the CTP decompensated group were significantly lower than those of patients in the CTP normal and CTP compensated groups (all *p* < 0.05). The RBANS total and its domain scores, including immediate memory, visual acuity, and delayed memory, in the CTP compensated and CTP decompensated groups were significantly lower than those in the CTP normal group (all *p* < 0.05). Multiple regression analyses showed that CTP compensation, CTP decompensation, severe VB tandem stenosis, and multiple infarctions were independent risk factors for cognitive impairment.

**Conclusions:** Low perfusion caused by severe VB stenosis can lead to extensive cognitive impairments in areas such as immediate memory, visual span, and delayed memory.

## Introduction

Neurocognitive function changes with age (1) and disease progression (2–4), which is related to pathologic mechanisms and is easily examined clinically. Carotid artery stenosis is closely related to vascular cognitive impairment (VCI) (5). Carotid artery stenosis can not only directly lead to the occurrence and rapid progression of VCI but also accelerate the development of degenerative diseases, such as Alzheimer's disease (6). Because of the collateral circulation in cerebral arteries, stenosis at the same site may cause different levels of cerebral blood flow perfusion. Studies have found that changes in cerebral flow perfusion were related to VCI in patients with carotid artery stenosis. Hypoperfusion caused by carotid artery stenosis can lead to frontal lobe damage, which in turn reduces the attention, language fluency, spatial structure, short-term memory, and executive function of patients (7). Compared with studies of VCI induced by carotid artery stenosis, few studies have examined the contribution of the posterior circulation or vertebra-basilar (VB) artery stenosis to cognitive impairment. Additionally, the correlation between cerebral blood flow perfusion and VCI in patients with VB artery stenosis remains unclear.

The stroke recurrence rate of the VB artery is reported to be relatively high (8, 9). For strokes in the posterior circulation or VB artery, transient ischaemic attack (TIA) accounts for ~20% of ischaemic stroke cases (10). The clinical presentation of posterior circulation ischaemic strokes is unapparent and differs from those of anterior circulation or carotid artery strokes. Consequently, this type of stroke is often hidden (11). Manifestations such as vertigo, diplopia, and coughing while drinking water are generally ignored by patients. In contrast, anterior circulation symptoms, such as facial or limb paralysis, are often more likely to be noted (12).

Basilar artery stenosis may lead to poor attention, poor executive function, and long-term memory impairment in patients (13). In this study, computed tomography perfusion (CTP) was used to analyse the relationship between cognitive impairment and cerebral haemodynamic changes. We aimed to investigate the cognitive status of patients with chronic posterior circulation hypoperfusion, which, to our best knowledge, has received little systemic investigation.

## Materials and methods

### Subjects

**Figure 1:**
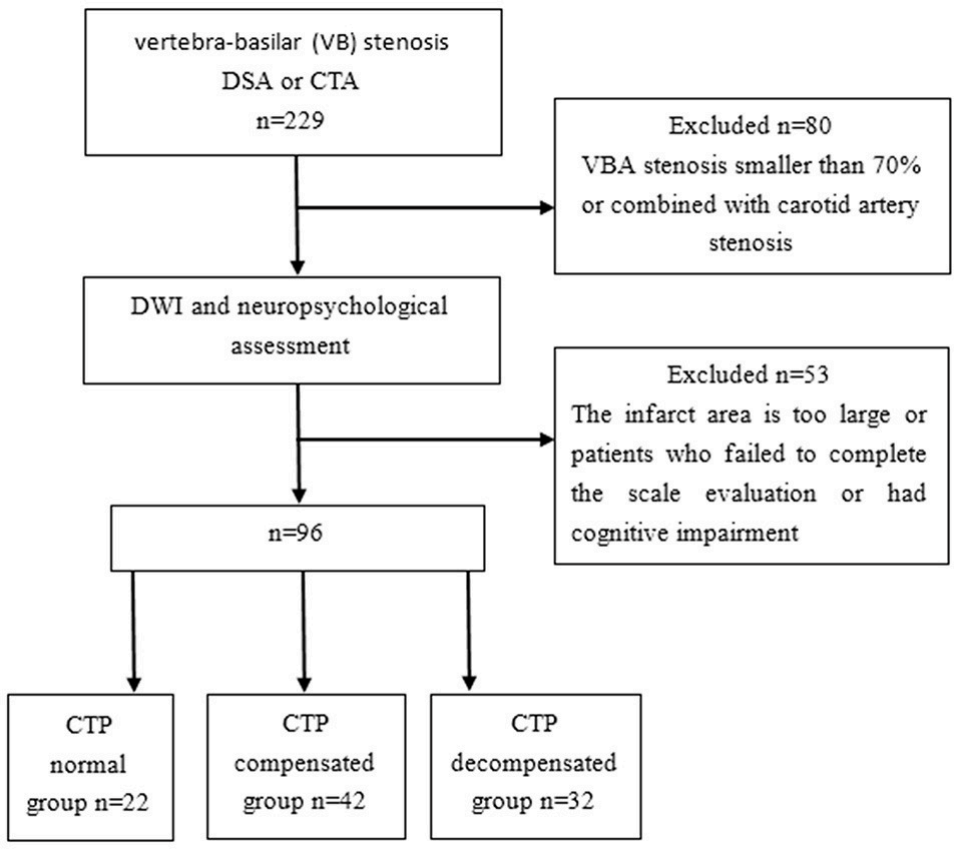
Recruitment diagram.

This study was a prospective cohort study (Clinical Trial Registration URL: http://www.clinicaltrials.gov. Unique identifier: NCT01968122.). All methods were performed in accordance with the relevant guidelines and regulations. A total of 96 patients who were diagnosed with severe posterior circulation VB stenosis and had infarction or a history of infarction for more than 2 weeks from January 2014 to January 2015 were enrolled in the current study. The inclusion criteria were as follows: (1) Patients who had vertebral artery or basilar artery stenosis confirmed by CT angiography (CTA) or digital subtraction angiography (DSA) examination, with a stenosis area equal to or greater than 70% of the vascular area (14–18). In this study, 70% of patients underwent CTA examination, 50% underwent DSA examination, and 20% underwent CTA and DSA examination. (2) Cranial magnetic resonance imaging (MRI) showed that the area of nonlacunar infarction [multiple infarctions, high signal greater than or equal to two diffusion-weighted imaging (DWI) images] was <1/3 of the hemisphere area. The exclusion criteria were as follows: (1) patients who failed to complete the scale evaluation due to aphasia, apraxia, and dysphonia; (2) patients who had cognitive impairment caused by Alzheimer's disease and other related nervous system degeneration or nonvascular factors; (3) patients who had nervous system diseases (such as central nervous system hereditary diseases, tumors, encephalitis, demyelinating disease, Parkinson's disease, craniocerebral injury, and epilepsy) that could lead to cognitive impairment; (4) patients who had anxiety, depression, or other mental disorders; (5) patients who had severe diseases of the liver, kidney, heart, or blood; (6) patients who had hypothyroidism, chronic alcoholism, infection, or other cognitive function-related diseases; (7) patients who had a history of substance abuse, drug addiction, carbon monoxide, pesticide, and other chemical poisoning, brain parasites, etc.; and (8) patients whose first-degree relatives had dementia and psychosis, cerebral lacuna infarct, or leukodystrophy revealed by brain MRI examination. The recruitment diagram is shown in Figure 1. After a complete description of the study, all subjects gave their written informed consent to participate in the study. This study was approved by the Regional Committee for Ethics of Beijing Tiantan Hospital.

### Baseline data assessment

The patients were divided into three groups: the CTP normal group, CTP compensated group, and CTP decompensated group (19). Baseline information, including gender, age, length of education, left- or right-handedness, high blood pressure, diabetes, atrial fibrillation, and smoking, was collected. Information on patient history of hypertension, diabetes, and hyperlipidaemia was recorded. Briefly, blood pressure ≥140/90 mm Hg (1 mm Hg = 0.133 kPa) was defined as hypertension, and fasting blood glucose ≥7.0 mmol/L, 2 h postprandial blood glucose ≥11.1 mmol/L or random blood glucose ≥11.1 mmol/L were defined as diabetes. Atrial fibrillation was diagnosed according to the 1979 World Health Organization (WHO) diagnostic criteria. Hyperlipidaemia was diagnosed based on the “Chinese Adult Dyslipidaemia Prevention and Control Guidelines” from 2007. Hyperlipidaemia was diagnosed when the patients met one of the following criteria: blood cholesterol concentration >5.17 mmol/L; blood concentration of triglycerides >1.7 mmol/L; or blood concentration of low-density lipoproteins >3.1 mmol/L.

### Cognitive function evaluation

Cognitive function was assessed using the validated Chinese version of the Mini-Mental State Examination (C-MMSE) (20), the Frontal Assessment Battery (FAB) (21), and the Repeatable Battery for the Assessment of Neuropsychological Status (RBANS) (22). Three researchers participated in a cognitive function training course before the study started. Repeated evaluations showed that the overall correlation coefficient of the MMSE, FAB, and RBANS for the three researchers was >0.8 after training. Finally, an RBANS total score >77.5 was defined as cognitively normal, and an RBANS total score ≤77.5 was defined as cognitively impaired (23).

### Imaging evaluation

Posterior circulation acute ischaemic infarction (including cerebral infarction) was diagnosed in patients with clinical manifestations. Additionally, such patients had high-density lesions on magnetic resonance DWI or TIA in the posterior circulation. CTP was performed using a Siemens dual-source spiral CT machine with 128 layers (Germany). Briefly, a volume of 60 mL of contrast agent (iohexol, 370 mg I/mL) was injected into the elbow middle vein at a rate of 8 mL/s using a double-tube high-pressure syringe (Ulrich Missouvi XD2501-C), and a volume shuttle scan with a scanning range of ~110 mm was started after a delay of 4 s. Intravenous injection of iohexol was performed using an EZEM high-pressure syringe (America) at a rate of 5 mL/s. The base section plane was selected, and two layers were continuously scanned 40 times with the parameters of 80 kV, 200 mA, layer thickness 12 mm, and pitch 0.75. Forty images in each layer were scanned, and a total of 80 images were obtained. Four images of the temporal lobe and 4 images of the occipital lobe were selected from each layer image as the region of interest (ROI). The original CTP image was introduced into a dedicated postprocessing workstation (Neusoft Medical Co., Shenyang, China) and analyzed with CT perfusion software. Time to peak (TTP), transit time (MTT), cerebral blood flow (CBF), and cerebral blood volume (CBV) were calculated. The qualitative assessment of perfusion in the ROI, which was used in a previous study (14), was grouped as follows. The patients in the CTP normal group had complete perfusion. The patients in the CTP compensated group had hypoperfusion and preserved cerebral vascular reactivity (a lower peak, delayed TTP, increased MTT, decreased CBF, and normal or elevated CBV). In addition, the patients in the CTP decompensated group had hypoperfusion without adequate cerebral vascular reactivity.

### Statistical analysis

All statistical analyses were performed using SPSS software version 23.0 (SPSS Inc., Chicago, IL, USA). Demographic and clinical variables of the multiple groups were compared using one-way ANOVA for continuous variables and X^2^ (chi-square test) or Fisher's exact test for categorical variables. Where there was significance in the ANOVA, we used the Fisher minimum significant difference (LSD) test for *post hoc* comparisons between groups.

A linear regression model was used to identify risk factors for cognitive impairment in patients with VB artery stenosis. *P* < 0.05 was considered statistically significant.

## Results

### Sociodemographic data and clinical background characteristics

A total of 96 patients were ultimately included in this study. Among them, 46 patients had severe basilar artery stenosis, 38 patients had severe intracranial artery stenosis, 32 patients had vertebral artery extracranial stenosis, 20 patients had tandem lesions, 12 patients had no new infarct (TIA), 56 patients had a single infarct, and 28 patients had multiple infarctions. The number of patients in the CTP normal group, CTP compensated group, and CTP decompensated group was 22, 42, and 32, respectively. There were no differences in the sociodemographic characteristics between the three groups (all *p* > 0.05). The rate of intracranial artery stenosis in the CTP compensated group was lower than that in the CTP normal group, (*p* < 0.05); however, the rate of intracranial artery stenosis in the CTP decompensated group was higher than that of the normal group (Table 1).

**Table 1:** Sociodemographic and clinical characteristics of the subjects (*n* = 96).

**Sociodemographic variables**	**Total cases (*n* = 96)**	**Group I (*n* = 22)^a^**	**Group II (*n* = 42)^b^**	**Group III (*n* = 32)^c^**	***P***
**SOCIODEMOGRAPHIC VARIABLES**					
Age, years	62.2 ± 11.8	60.9 ± 11.5	63.1 ± 12.1	61.9 ± 11.9	0.772
Male sex (%)	74 (77.1)	16 (72.7)	34 (81.0)	24 (75.0)	0.715
Education, years	7.3 ± 2.4	7.6 ± 2.6	7.2 ± 2.4	7.3 ± 2.4	0.800
Hypertension (%)	50 (52.1)	12 (54.5)	20 (47.6)	18 (56.3)	0.737
Diabetes mellitus (%)	36 (37.5)	8 (36.4)	14 (33.3)	14 (43.8)	0.652
Atrial fibrillation (%)	6 (6.3)	0	4 (9.5)	2 (6.3)	0.327
Cigarette smoking (%)	56 (58.3)	14 (63.6)	24 (57.1)	18 (56.3)	0.845
Hyperlipidaemia (%)	46 (47.9)	8 (36.4)	18 (42.9)	20 (62.5)	0.114
**LESION SITE**					
Basilar artery stenosis (%)	46 (47.9)	6 (27.3)	22 (52.4)	18 (56.3)	0.083
Intracranial artery stenosis (%)	38 (39.6)	6 (27.3)	18 (42.9)	14 (43.8)	0.404
Extracranial artery stenosis (%)	32 (33.3)	16 (72.7)	6 (14.3)^*^	10 (31.3)	0.000
Tandem lesion (%)	20 (20.8)	6 (27.3)	4 (9.5)	10 (31.3)	0.052
**INFARCT PATTERN**					
No new infarct (%)	12 (12.5)	4 (18.2)	6 (14.3)	2 (6.3)	0.550
Single infarct (%)	56 (58.3)	12 (54.5)	26 (61.9)	18 (56.3)	
Multiple infarction (%)	28 (29.2)	6 (27.3)	10 (23.8)	12 (37.5)	

a Group I, CTP normal group;

b Group II, CTP compensated group;

c Group III, CTP decompensated group.

* *P < 0.05, compared with group I*.

### Association between MMSE, FAB, and RBANS scores and CT perfusion

**Figure 2:**
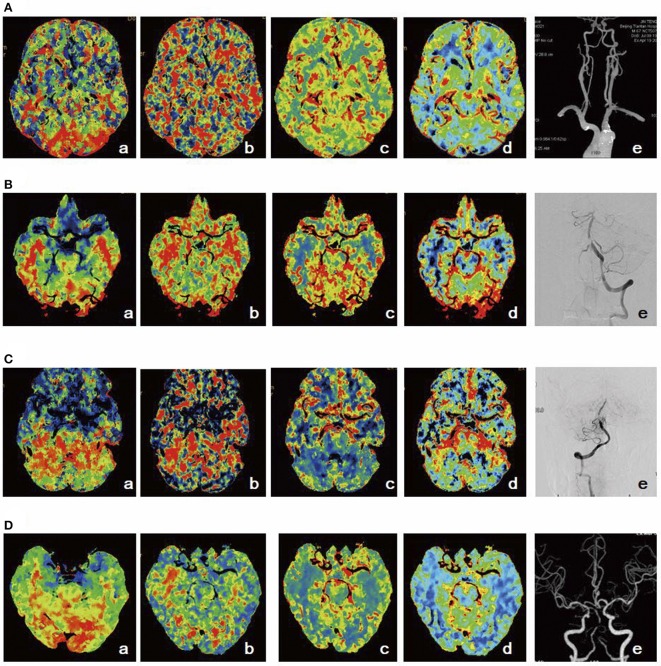
Schematic diagram of posterior circulation perfusion. **(A)** Stage I1: TTP was prolonged (a), MTT (b), CBF (c), and CBV (d) were normal, and CTA suggested severe stenosis of the left vertebral artery opening (e). **(B)** Stage I2: TTP (a) and MTT (b) were prolonged, CBF was slightly decreased (c), CBV was elevated (d), and DSA indicated severe proximal stenosis of the basilar artery (e). **(C)** Stage I3: TTP (a) and MTT (b) were prolonged, CBF was decreased (c), CBV was slightly decreased (d), and DSA indicated severe stenosis in the middle part of the basilar artery (e). **(D)** Stage I4: TTP (a) and MTT (b) were prolonged, CBF was decreased (c), CBV was decreased (d), and CTA demonstrated occlusion in the middle part of the basilar artery (e).

The stages of posterior circulation perfusion are summarized in Figure 2 in the order of cognitive decline.

As presented in Table 2, the MMSE, FAB, and RBANS scores of the CTP decompensated group were significantly lower than those of the CTP normal and CTP compensated groups (all *p* < 0.05). The RBANS total, immediate memory, visual acuity, and delayed memory scores in the CTP compensated and CTP decompensated groups were significantly lower than those in the CTP normal group (*p* < 0.05). CTP compensated patients had reduced attention compared to that of CTP normal patients (*p* < 0.05).

**Table 2:** The association between MMSE, FAB, and RBANS scores and CT perfusion.

**Cognitive evaluation**	**Group I^a^**	**Group II^b^**	**Group III^c^**	***p***
MMSE score	25.29 ± 3.16	24.14 ± 1.73	20.37 ± 3.89^*^^Δ^	0.000
FAB score	16.25 ± 3.44	15.46 ± 4.12	13.08 ± 4.72^*^^Δ^	0.013
RBANS score	80.84 ± 14.77	70.80 ± 9.65^*^	58.94 ± 11.14^*^^Δ^	0.000
Immediate memory	85.54 ± 11.09	68.76 ± 18.33^*^	58.85 ± 14.52^*^^Δ^	0.000
Visuospatial/constructional function	83.23 ± 15.80	71.59 ± 14.68^*^	62.96 ± 15.00^*^^Δ^	0.000
Language	82.00 ± 9.61	81.87 ± 10.07	79.74 ± 10.64	0.614
Attention	87.97 ± 9.21	73.40 ± 14.52^*^	76.18 ± 12.74^*^	0.000
Delayed memory	87.33 ± 10.88	71.68 ± 12.32^*^	62.91 ± 13.9^*^^Δ^	0.000

a Group I, CTP normal group;

b Group II, CTP compensated group;

c Group III, CTP decompensated group.

* P < 0.05, compared with group I.

Δ *P < 0.05, compared with group II. MMSE, Mini-Mental State Examination; FAB, Frontal Assessment Battery; RBANS, Repeatable Battery for the Assessment of Neuropsychological Status*.

### Regression models of independent risk factors for cognitive impairment

Based on the RBANS total score, 68 patients were included in the cognitive impairment group (RBANS score ≤77.5), and 28 patients were considered to have no cognitive impairment (RBANS score >77.5 points). After adjusting for other relevant factors, CTP compensation (*p* = 0.30), CTP decompensation (*p* < 0.01), severe VB tandem stenosis (*p* = 0.021), and multiple infarctions (*p* = 0.023) were found to be independent risk factors for cognitive impairment (Table 3).

**Table 3:** Regression models of independent risk factors for cognitive impairment.

	**Cognitive impairment**	**No cognitive impairment**	**Regression coefficient**	***t***	***P***
Compensation (%)^a^	34 (50)	8 (29)	3.313	2.241	0.030
Decompensation (%)^a^	30 (44)	2 (7)	6.425	4.415	<0.001
Series or multiple stenosis (%)^b^	16 (24)	4 (14)	3.524	2.573	0.021
Multiple infarction (%)^c^	20 (30)	8 (22)	3.276	2.689	0.023

a Adjusted for age, sex, hypertension, diabetes, atrial fibrillation, cigarette smoking, hyperlipidaemia, lesion site, and infarct pattern;

b Adjusted for age, sex, hypertension, diabetes, atrial fibrillation, cigarette smoking, hyperlipidaemia, perfusion type, and infarct pattern;

c *Adjusted for age, sex, hypertension, diabetes, atrial fibrillation cigarette smoking, hyperlipidaemia, perfusion type, and lesion site*.

## Discussion

The primary findings of this study could be summarized as follows: (A) patients who have chronic posterior circulation hypoperfusion showed a decline in cognitive ability; (B) medial temporal lobe perfusion was associated with serious cognitive impairment; (C) in addition to language ability, there were other dimensions of cognitive impairment; and (D) in patients with chronic posterior circulation hypoperfusion, multiple stenosis and multiple infarcts were independent risk factors for cognitive impairment.

The basilar artery branches into two posterior cerebral arteries (PCA), which supply the majority of blood to the temporal lobe and thalamus. Previous studies found that cognitive impairment existed in patients with infarcts in these regions (24–26). In our study, the cognitive ability of patients with low-perfusion percutaneous coronary intervention (PCI) generally decreased, which might be associated with chronic ischaemia and hypoxia of the brain structures mentioned above. Studies showed that the state of ischaemia and hypoxia was associated with damage to the neural network between the brainstem or cerebellar regions and the anterior circulation (25–27). Low perfusion leads to a decrease in thrombus clearance; additionally, the formation of microemboli that result from lesions caused by cerebral vascular stenosis also leads to VCI (28). In animal studies, microemboli were found to decrease the number of brain-derived neurotrophic factors in the hippocampus and lead to impaired memory in mice (29).

In our study, the executive function, immediate memory, delayed memory, and visual range of patients with PCI accompanied by hypoperfusion were impaired, which is in agreement with previous findings (30, 31). However, the language function of these patients was retained in our study, which is inconsistent with previous studies (31, 32). In these patients, the memory function, including short-term memory and delayed memory, was severely damaged, which might be related to long-term ischaemia and hypoxia of the medial temporal lobe structures. The efferent fibers and afferent fibers of the temporal lobe have a wide range of links with the frontal lobe, parietal lobe, occipital lobe, and hippocampus (33). The hippocampus plays an important role in mood, neuropsychological activities, memory, execution, language (including fluency and repetition), and other cognitive activities (34). Memory impairment may occur before stroke, which might be associated with the chronic ischaemia and hypoxia caused by the hypoperfusion of the medial temporal lobe (34). Executive function impairment may be caused by damage in part of the tissues of the VB artery, whose function is linked to the thalamus, parietal lobe, and frontal lobes (25, 26, 35, 36). Visual span impairment might be associated with chronic ischaemia and hypoxia in the occipital lobe and temporal lobe (19, 37). Further multifactor logistic regression analysis revealed that low perfusion of blood supply areas, tandem, or multiple stenosis, and multiple PCI were independent risk factors for cognitive impairment in patients with PCI. Both CTP compensated and CTP decompensated patients had cognitive impairment. The incidence of cognitive impairment in CTP decompensated patients was 6.8 times higher than that observed in the normal metabolic patients. A previous study found that the prognostic MRS score of patients with PCI was significantly higher than that of patients with anterior circulation infarction (38). Although the neurological function of patients with PCI recovers well, their cognitive function is likely to suffer sustained damage if the collateral circulation is not sufficient or chronic hypoperfusion is persistent. Tandem lesions or multiple stenosis can lead to a further decrease in perfusion in the posterior circulation area (39). The presence of chronic persistent hypoperfusion can lead to multiple infarcts in the brain, which also aggravates the cognitive impairment of patients (40). It was reported that in first-onset mild stroke patients, the occurrence of multiple infarcts and decreased hippocampal volume were positively correlated with cognitive impairment (41). Even in patients with asymptomatic stroke, multiple infarcts caused by hypoperfusion or microemboli also led to reduced hippocampal volume, resulting in decreased memory. In addition, multiple cerebral infarctions led to declines in language function, processing speed, and visual spatial competence (42).

The present study has some limitations. First, the RBANS was performed by only a single independent reviewer; therefore, there was some subjectivity in the judgement of graphic memory. Second, a small number of patients had carotid artery stenosis. As a result, these patients may be affected by cognitive effects due to anterior circulation cerebral hypoperfusion. Third, the sample size of the study is relatively small, which limits the generalizability of the results. Hence, the conclusions must be further confirmed with a larger sample size. In the future, we will design different experiments related to neurocognitive function (43, 44) and use various analytical methods to explore the pathologic mechanisms of neurocognitive deficits in patients.

## Author contributions

YD and LW: analyzed and interpreted the data, wrote the paper. XS, LL, MZ, and CW: contributed to the conception or design of the work, interpreted the data. BS, MS, WG, and DM: conceived and designed the experiments, performed the experiments. NM, LS, XL, ZM, and XH: performed the experiments, drafted and revised the work. DC and FG: revised the paper, approved the final version.

### Conflict of interest statement

The authors declare that the research was conducted in the absence of any commercial or financial relationships that could be construed as a potential conflict of interest.

## Abbreviations

VB: Vertebra-Basilar
CTP: Computed Tomography Perfusion
MMSE: Mini-Mental State Examination
FAB: Frontal Assessment Battery
RBANS: Repeatable Battery For The Assessment Of Neuropsychological Status
VCI: Vascular Cognitive Impairment
TIA: Transient Ischaemic Attack
CTA: Ct Angiography
DSA: Digital Subtraction Angiography
DWI: Diffusion-Weighted Imaging
MRI: Magnetic Resonance Imaging
TTP: Time To Peak
MTT: Transit Time
CBF: Cerebral Blood Flow
CBV: Cerebral Blood Volume
PCA: Posterior Cerebral Arteries
PCI: Percutaneous Coronary Intervention.
